# Healthcare students’ perceptions about their role, confidence and competence to deliver brief public health interventions and advice

**DOI:** 10.1186/s12909-018-1224-0

**Published:** 2018-05-24

**Authors:** Sionnadh McLean, Laura Charlesworth, Stephen May, Nick Pollard

**Affiliations:** Faculty of Health and Wellbeing, Sheffield Hallam University, Robert Winston Building, Sheffield, S10 2BP UK

**Keywords:** Public health messages, Allied health professionals, Students, Qualitative study

## Abstract

**Background:**

Public health improvement has long been an important focus for the United Kingdom Department of Health. The Allied Health Professions (AHP) Federation has 84,000 members, such a large number of AHP professionals should play a role in public health initiatives, but it is not clear if they or the AHP students who will be the future healthcare workforce feel themselves equipped to do so. Our aim was to understand the perceptions of AHP students about their role in delivering public health advice.

**Methods:**

AHP students were recruited in one teaching university from different departments. Participants were final year AHP students who had completed all clinical placements related to their course. All students were emailed an invitation to participate, and those interested were asked to contact the researchers to participate in one of several focus groups. Data were recorded, transcribed, and analysed using framework analysis by two independent researchers.

**Results:**

Nineteen students were recruited and participated in four focus groups. The main themes produced by the data analysis were: understanding of public health issues, perceptions of their role in this, challenges and opportunities to develop a public health role, and preparation for a public health role.

**Conclusions:**

AHP students felt that they had a role in public health advice-giving, but barriers to providing this advice included their own lack of confidence and knowledge, time, and the environment of the clinical placement. They considered that there should be more teaching on public health issues, and that these should feature in both the curriculum and on clinical placement.

**Electronic supplementary material:**

The online version of this article (10.1186/s12909-018-1224-0) contains supplementary material, which is available to authorized users.

## Background

Public health improvement has long been an important focus for the Department of Health and there is a growing focus in the United Kingdom for the contribution of the wider public health workforce, including the Allied Health Professions to the public health agenda. Public health services in England were restructured and Public Health England was established on 1 April 2013 to bring together public health specialists from more than 70 organisations into a single public health service to work alongside other healthcare services to deliver and improve public health services [1]. Its aim is to improve the health of the nation and to reduce health inequalities throughout England. Helping people to live healthy lives and reduce disease burden of health risks associated with smoking and alcohol, obesity, poor diet, poor mental health and physical inactivity is a priority area [2]. The Department of Health and the National Health Service have produced numerous documents highlighting the importance of improving health. The white paper “Saving lives: Our healthier Nation” provided a national action plan to reduce health inequalities and reduce incidence of cancer and strokes in the United Kingdom (UK) [3]. The basis of the paper identifies that in some cases healthy living is a lifestyle choice, and that individuals can increasingly be encouraged to make positive lifestyle choices with the provision of appropriate information, which may lead to reduced mortality. However, the implementation of health improvement did not follow. The concept of health improvement proved complex, and unclear goals and priorities, such as whether children or adults should be the targets of obesity prevention, created confusion [4]. The impact of guidelines on individual clinical practice may not have an effect without the backing of trust and departmental implementation strategies, for instance staff may not see it as a clinical priority due other pressures [5]. The key messages that followed the 1999 white paper were that public health issues must be tackled by partnerships of health and social care services, and that each member of the health service can provide public health messages which lead to overall improvements to health and do not just deal with the recognised health problems [6–9]. There is a growing focus in the United Kingdom for role the Allied Health Professions in improving population health and embedding prevention throughout all aspects of healthcare and subsequently the Allied Health Professionals Federation and Public Health England have identified the need for a strategy to support the delivery of public health messages within AHP practice. The publication of the strategy to develop the capacity, impact and profile of AHPs in public health 2015–2018 identifies the need for strategic leadership and support from partner organisations to embed this strategy into practice [10].

The Allied Health Professions Federation represents 84,000 members of the AHP workforce (all health professions with the exception of nurses, midwives and physicians), around 1 in 5 of the total number of National Health Service employees. AHP commitment to public health is confirmed with statements for actions from several professional bodies [11–13], AHPs directly work with the public, highlighting their great potential to deliver key messages [14] which are appropriate to patients. The success of these policies requires brief public health messages to be given on a regular basis and in a consistent manner.

Whilst primary care services provide appropriate platforms, for example enhanced support and integrated service models [15], several studies suggest that there are numerous barriers and challenges to implementation of such activities. This includes poor recognition by healthcare professions of their role in opportunistic health promotion delivery, lack of time, limited knowledge and expertise in areas of health promotion delivery [16, 17]. In order to ensure the competence of future AHP workforces, Higher Education Institutions (HEI) have an important role to develop a sound knowledge base in health improvement and promotion in all undergraduate and postgraduate health degree courses [13, 14]. As far as we are aware, the confidence and competence of AHP students to deliver on Public Health has not been investigated and therefore the aim of this study was to conduct an initial investigation into the perceptions of AHP students regarding their role, confidence, competence, barriers and challenges to delivering brief public health interventions and advice.

## Methods

### Research design

The project employed a qualitative design to explore the perceptions of AHP students in one teaching University. Focus groups were conducted by a facilitator using an interview schedule to gain insights into participants’ perceptions regarding their role, confidence, competence, barriers and challenges to delivering brief public health interventions and advice (BPHIA). Four focus groups were conducted in two closely grouped pairs with a two week interval to allow time for transcription, analysis and refinement of the topic guide (Additional file 1) therefore enabling the facilitator to explore relevant issues more deeply at the subsequent pair of focus groups [18].

### Ethics, participants and recruitment

The project received ethical approval from the Sheffield Hallam University research ethics committee. An invitation was sent by email to all AHP at Sheffield Hallam University inviting them to contact the lead researcher. Students who met the inclusion criteria (final year AHP students who had completed all clinical placements for their course) and who indicated their willingness to be involved were asked to sign a consent form and complete a basic questionnaire outlining their clinical background and demographic details, before invitation to attend a focus group at a convenient time and venue. To ensure full exploration of issues recruitment continued until data saturation had occurred, identified by recurring themes and no new insights emerging through further data collection.

Two researchers reviewed and coded the data, discussed themes and clarified the coding framework, themes, subordinate themes and supporting extracts. Participants were given the opportunity to review transcripts, themes and interpretations, and make additions or corrections which ensured the accuracy of the information and protect against potential misinterpretations and researcher subjectivity [18, 19].

### Data collection and analysis

Four focus groups were conducted and audio recorded at Sheffield Hallam University, each led by one of the authors. An interview schedule (Additional file 1) was used to explore the perceived, knowledge, confidence and competence of participants to deliver brief public health interventions and advice.

Analysis was conducted using the framework approach to identify concepts, categories and themes in the data [20]. This method was developed for the purpose of conducting applied qualitative research and is an excellent method for investigating healthcare policies and procedures or the experience of those affected by policy decision. It also provides systematic and visible stages of data analysis and allows for integration of pre-existing themes into the emerging data analysis [21]. The five key stages of framework analysis are: familiarization, identification of a thematic framework, indexing, charting, mapping and interpretation. An initial thematic coding framework was developed based on the topic guide (Additional file 1) and then modified following preliminary analysis of the transcripts from the first pair of focus groups. The revised coding framework was used to analyse all the transcripts. Data from all the focus groups were charted and summarised in separate documents and similar responses to questions were grouped together to identify themes.

## Results

Saturation (identified by recurring themes and no new insights emerging through further data collection) was observed after three focus group sessions; to confirm this one additional focus group was conducted; each consisting of five to ten participants per group, and lasting for approximately two hours. The sample (*n* = 19) consisted of 17 female and 2 male students (see 1.0 yrs. (Table 1).

**Table 1:** Allied Health Profession Representation

Allied Health Profession	Male	Female
Physiotherapy	2	7
Diagnostic Radiography	0	4
Therapeutic Radiography	0	3
Occupational Therapy	0	3
Total:	2	17

Four main themes and 12 sub-themes were derived from the focus groups interviews (see Table 2 and Additional file 1). Students freely discussed issues relating to 1) their understanding of public health, 2) the perceptions around their role and the role of others, 3) the barriers and challenges of integrating public health matters into their clinical role and 4) preparation for a public health role.

**Table 2:** Themes and sub-themes derived from focus groups

Theme	Sub-themes
1. Understanding PH	Who is driving public health
	What are the key public health issues
	Knowledge of named campaigns
2. Perception of Role	Role of different professional groups
	Perception of responsibility
3. Challenges and opportunities	Provision of PH referral pathways or appropriate services
	Therapeutic relationships and communication
	Time
4. Preparation for PH role	Academic preparation
	Clinical placement
	Personal responsibility to prepare for PH role

## Understanding public health

Students from all disciplines were aware that public health relates to the promotion and protection of societal health and well-being.

“I’d say it [public health] is the wider health issues that [concern] everybody, rather than necessarily what they’re specifically seeing you on that occasion for, it’s their general wellbeing and health that is part and parcel”. FG2, p1, l31-37

They were familiar with key public health issues including smoking, obesity, physical inactivity, alcohol and drug abuse. There were varying levels of awareness of a wider range of public health issues including sleep quality, dealing with stress, screening and vaccinations, cancer awareness and screening and mental health disorders. Students were aware of the existence of numerous national public health campaigns, such as “Change for Life”, smoking cessation programmes and smoking bans, MOT (overall health) checks, “be clear on cancer”, “the walking bus” and healthy eating campaigns such as “Five-a-day”. They demonstrated weak understanding of the content and key messages emerging from the majority of these campaigns.

“Being active, obesity, alcohol management, drugs and mental health as well. I think a lot of the choices that you decide to make can obviously affect your health, and it’s from then that you might need to seek more help from services.” FG 1, p2, l3-6

Students did not understand the nature of brief public health interventions and advice i.e. short intervention periods of around 30 s or so which involve opportunistic advice, discussion, negotiation or encouragement on a variety of key public health issues. They were also uncertain of the agents who are driving the public health agenda in the UK. They reported no knowledge of Public Health England, though some students realised there would be government support for public health. Students were very uncertain about the role of their own professional bodies in public health and none were aware of any public health guidelines provided by their professional bodies.

## Perception of role

All students agreed that all health professions, including medical and nursing groups, have an important role in delivering public health messages and signposting patients to appropriate services. They felt that the different professional groups have different roles, skills, knowledge and experience which make it easier to deliver certain messages; for example, physiotherapists and exercise, or therapeutic radiographers and smoking.

Therapeutic radiography students presented conflicting views about their role. Most suggested that they have such little time with their patients that delivering their highly technological interventions is the greater priority. One student suggested that radiotherapy may attract more technical and less empathetic individuals with lesser capacity to deliver public health messages. On the other hand, therapeutic radiography students provided several examples of delivering public health messages on placement and could also see a particular role in delivering smoking cessation messages. Students viewed doctors and nurses as more medically and technically focussed, which limited their capacity and opportunity to deliver public health messages. All students felt that in practice none of the professional groups were delivering public health messages consistently or effectively.

“F: I think anyone that comes in contact with say the public as a health profession we’ve all got a duty of care to make sure that we’re doing our bit to either promote health or help give them advice or something like that” FG 3, p4, l1-4

“F: Probably quite limited I think for radiotherapy….. I’d say the main intervention we offer as therapy radiographers is smoking cessation because smoking during radiotherapy can make your side effects a lot worse.” FG 2, p7, l8-17

Students recognised that the ability to refer to other appropriate public health services or pathways of care was important. Students saw patients, and in some cases families and carers, as having a responsibility to be ready to listen and willing to make a change.

The majority of the occupational therapy and physiotherapy students saw themselves as key players in promoting a wide range of public health messages, however students felt the need for some discretion about how they approach issues with patients, and that they had a personal role in making public health messages patient-friendly.

“F…. we’re here to signpost services for patients. We’re not there to preach or to bang on about the same thing day-in day-out. That’s where a bit of like discretion or, we all know when a patient is not being receptive to what you say, so you just pack it in don’t you?” FG 2, p14, line 19-30

## Challenges and opportunities

A range of factors impacted on student confidence and competence to deliver public health messages.

- Provision of referral pathways or appropriate services

Provision of BPHIA is enhanced where adequate referral process are in place. Students identified some of the local public health services available but recognised the need for enhanced personal knowledge of local services and a better understanding of referral processes to support the delivery of BPHIA. Links between professional groups (from AHP to general practitioner) are important in ensuring follow through of the intervention.

" … all my placements have been in Sheffield and that’s actually been really useful because I know quite a few services and groups that are available here……. it’s so crucial to have that knowledge about what’s available for your patients…. " FG 1, p28 l34-p29 l6

Students thought it would be beneficial to be taught how and where to refer or signpost patients to public health services. Student confidence and delivery is increased in areas of practice with referral processes and is absent in areas where referral processes do not exist; or, as one diagnostic radiography student claimed, are not recognised.

"And we don’t have the training to refer either ….. we don’t have the knowledge of who to refer to". FG 4, P 23 l 8-11

b. Relationships and communication

Students expressed concerns in two key areas: patient-therapist and inter-disciplinary relationships and communication. All students identified being “*worried about jeopardising the relationship” (FG3 P5 l19–20)* between patient and therapist, particularly on difficult topics such as smoking, weight, alcohol and drug use. They felt that building a rapport and trust with the patient around their current health problem took precedence. Students felt more confident about broaching the conversation about public health where they felt more knowledgeable or where there was a direct relationship with the patient’s health problem. For example, therapeutic radiographers were most confident to discuss smoking cessation, because it has a direct consequence on patients’ symptoms during radiotherapy treatment; physiotherapists were most confident about discussing exercise and physical activity, because it is a recognised way of managing most health problems. Students identified the importance of inter-disciplinary public health pathways that were connected and consistent. Referrals and signposting were only considered worthwhile if the follow-up services also carried out their role. Students also expressed concerns over how the views of a newly qualified AHP might be received within the multidisciplinary team.

Confidence was reduced for topics which they considered difficult to broach, for instance, alcohol dependency or obesity, or knowing how to offer public health advice if it was unrelated to current treatment.

"I wouldn’t say I feel very confident giving people public health information, like dietary stuff I wouldn’t really say I know about." FG 2, P30, l 18-21

c. Time and competing priorities

Although students recognised and identified some areas of good practice and delivery most students felt that there was inconsistency in the implementation of BPHIA. Time and competing priorities were viewed as a barrier by all students though this varied across different professional groups. While students felt that time was required to build sufficient rapport before tackling difficult issues, departmental culture and the support received from staff and educators was also seen to influence their delivery of BPHIA.

"I think it depends on the setting as well, because if you’re a physio in a group like a rehab class you always have some education time so you usually do things like that in your education, but if it’s an outpatient appointment when you’ve got 20 minutes, so you’re not wasting time on whether they smoke or not, because it’s just a different setting as well." FG 3, P15, l 28-32

Diagnostic radiography students in particular felt there was insufficient time to build a rapport and discuss lifestyle factors, when their priority was delivering more technical treatments. Occupational therapists thought that because they frequently spend longer with patients than other AHPs they could find time to discuss BPHIA interventions, however all students recognised that AHPs may have more time than doctors and nurses to deliver on BPHIA.

## Preparation for public health role

To varying degrees, all the AHP students felt a personal responsibility to engage in continuing professional development to develop their capacity to deliver on the public health role and that it was crucial to have a better understanding of the key public health messages. They felt that broadening out their healthcare delivery to include public health issues would lead to better holistic care of their patients. They felt that they were responsible for finding out about key public health messages they could be delivering and about public health services that were available to support their clinical environment. However they also felt that education provided through academic preparation and clinical placements were vital.

All students felt that they had received some academic preparation which had increased their awareness and knowledge about public health issues. Physiotherapy students had undertaken a public health qualification in year 1 of their course. Occupational therapy and physiotherapy students described undertaking modules and assignments that related to public health issues. However many students felt that the public health issues were becoming increasingly central to clinical practice, and that this element should be more pronounced in AHP courses. With regard to their variation in confidence about broaching public health issues, all students felt that the university could offer more instruction on negotiating difficult topics and delivering BPHIA without offending or “nagging” patients.

“actually health promotion in OT is becoming a very big thing, and it’s something that OTs could have a much bigger role in to meet the needs of the public. Whereas my teaching […] was only a very small three day module. And really considering now the role that we could have, it should be so much bigger…”. FG 1, p18 l31 - p19l 8*.*

*“F: And it’s like you said you didn’t feel confident to say anything about patients with OA because you didn’t know that that was definitely the right thing, so I think if they made an effort to teach us that side of things as well”* FG3, *pp19, line 36 to pg 20, l 37*

Therapeutic and diagnostic radiography students felt that public health issues were not seen as being within the scope of practice and stated that they did not observe BPHIA in action and were not encouraged to implement it themselves by their clinical educators. Physiotherapy and occupational therapy students found the opportunities to apply BPHIA whilst on clinical placement were variable, reporting that some placement supervisors encouraged the application of public health issues. Although all participants could identify the potential for BPHIA, students attributed their lack of confidence to do this due firstly to a lack of public health content in academic modules, and second to lack of encouragement or support by placement educators.

“I wouldn’t say I've ever been encouraged really. I can’t ever think of a moment on all of my six placements that I've been told to, or encouraged to deliver a certain public health message”. FG 1, p24, l27-32

## Discussion

This study identified four key themes namely: understanding of public health issues; perceptions of their role in this; challenges and opportunities to develop a public health role; and preparation for a public health role. These themes give rise to four key areas which are discussed below.

### Conflicting perceptions of role

Participants recognised that they had an important role in delivering BPHIA, however radiography students did not observe any clinical exposure to BPHIA during clinical practice.

These findings are supported by research previously undertaken in the recent healthy conversations paper [22]. The healthy conversations project surveyed qualified allied health professionals surrounding their role in public health, and found that 87.6% of individuals agreed that their role should include an element of preventative ill health. However a recent extensive meta-ethnography [16] suggests that health care professionals’ willingness to engage in public health related activities depended upon whether they were biopsychosocially or biomedically oriented. Primary care health professionals with a more biomedical orientation were less likely to engage in disease prevention and promotion of healthy lifestyles, thought such activities were peripheral to their field of work and was the responsibility of community or Government. On the other hand, healthcare professionals that adopted a biopsychosocial approach thought that public health activities were an important part of their role and felt responsible for implementing these activities in practice. This literature review almost exclusively presents findings related to primary care physicians and nurses and therefore may not accurately reflect the views of AHPs [16]. It is possible that recent promotion of the role of AHP’s in public health may be increasing the positive attitudes and BPHIA of AHP staff. However, according to our study this was not reflected in the student’s clinical placement experiences, where there was generally conflicting, but low levels engagement of AHPs with BPHIA.

### Barriers to delivery of BPHIA

Students identified a range of barriers to the delivery of BPHIA, including environment, culture, time and variations in the practice of qualified AHPs. These barriers and challenges to delivering public health messages were also identified by physicians and nurses working in primary care [16]. Often students felt that placement experiences did not offer a safe environment to broach difficult topics. Support from clinical staff and educators positively influenced the delivery of BPHIA, and more should be done to ensure safe opportunities are provided for students during clinical placement. Students strongly agreed that the delivery of BPHIA was part of their role however lacked confidence and were inhibited where this was not encouraged by clinical staff and educators. Although students agreed that BPHIA was part of their role they were unclear about what their exact role should be. Students identified their thoughts regarding priority areas for different professional areas; however students were unaware of professional body guidance and were mostly unable to refer to academic teaching or clinical experience to identify their specific professional role in the delivery of BPHIA.

The participants’ views concerning time as a barrier to provision correlate with those of practising AHPs in the literature [23]. One possible explanatory factor may be students’ limited knowledge of BPHIA which they attributed to lack of coverage in education. If so, the qualified AHP workforce in the evidence base may have experienced a similar deficit. The sense that there may be insufficient time to deliver BPHIA may result from limited knowledge and training around the delivery of brief interventions. Timeliness and the appropriateness of delivery in the patient pathway were also identified as a barrier and an area of concern. Students who felt confident to deliver BPHIA were concerned about how to manage the complex conversations that make take place following the brief intervention, with a lack of infrastructure to support referral pathways. They had limited experience of observing strategies to overcome this in the clinical setting.

Across the AHP disciplines many health topic areas are recognised as ‘difficult conversations’. Throughout the AHP academic and clinical training students are supported with skill development to hold difficult conversations, such as terminal illness, progressive illness and disability; but students did not recognise that this skill is transferable to other areas, including public health.

The clinical environment has a clear role in developing student confidence and competence in the delivery of BPHIA and can act as a barrier or facilitator to their contribution to the public health agenda. If the workplace does not encourage this, students are likely to avoid broaching difficult conversations, limiting experience and knowledge gained through placement opportunities. Students also recognised the significance of adequate referral processes. Although some students were able to identify some local services, a clear link between an absence of referral process and absence of delivery of BPHIA was established. The need for a clear clinical infrastructure to fully embed referral pathways within practice is necessary to further support the role of AHPs in public health and this is identified as a recommendation for practice. There is a risk that ineffective health communication can generate harm, loss of confidence in services, and even lead to death [24].

### Building confidence and competence in the future AHP workforce

Students identified some teaching of public health concepts during academic blocks in the university, which varied across professions. However there was a clear gap in knowledge surrounding ***brief*** public health interventions and advice. In order for students to develop confidence in the delivery of BPHIA several areas for development have been identified; firstly, in addition to the delivery of public health content in the academic setting students requested teaching that is focused on *how* to deliver BPHIA. Knowledge of public health topics *only* was not sufficient for students to translate this into the delivery of BPHIA in a clinical setting. Secondly, lack of confidence can be explained through limited experiential learning around broaching difficult topics with service users in practice. Although students received teaching on how to engage in difficult conversations within many AHP undergraduate courses this was not always clearly identified as a transferable skill to other difficult conversations such as BPHIA.

A further inhibiter to student confidence can be explained by limited opportunities to practise the delivery of BPHIA in a safe and supportive environment. The clinical placement setting must provide students with the opportunity to broach such conversations with service users and to provide mentor support to enable students to develop confidence in this area. Student experience of clinical placement support varied considerably across the professions and even across rotations within individual professional groups.

The importance of the placement setting in the delivery of public health during pre-registration AHP courses is identified by the Council of Deans of Health [25]. This review of current practice recognises the significant amount of time that learners spend on clinical placement, recognising the need for students to translate knowledge into practice. This research identifies a clear need to develop the curriculum to include a core public health element within undergraduate AHP courses, although embedding further content will be a challenge given other demands for better clinical knowledge [25]. One current issue is the quantity and content of currently delivered public health material and how this should be standardised across AHP courses. The Council of Deans of Health [25] identified eight key areas of public health; this could be used as an initial framework, with subject specific content embedded where appropriate for individual courses.

Further research toward wider assessment of HEI’s and the evidence base to develop a robust finalised framework for public health within the AHP curriculum has been recommended, which should include the role and contribution of clinical partners [25]. Further research with clinical staff to understand their contribution to the public health agenda and how significant individuals feel that public health is to their individual job role would enable broader understanding of the perceptions of AHP students in this study. This would also provide information surrounding gaps in knowledge that could be supported through the development of a postgraduate framework for public health to enable future support from the clinical workforce in the integration of public health within the clinical and academic setting. The development of the clinical environment not only requires support for knowledge and confidence of staff and students to deliver BPHIA but also the need for a robust clinical infrastructure to support the delivery of public health in AHP settings. This includes the need for support from an organisational level to provide referral pathways that are effective for service users.

### Strengths and limitations

This project provides a unique contribution to the evidence base, and focused on final year AHP students’ perceptions regarding their role in the delivery of brief public health interventions and advice. This viewpoint is essential as the student group represents the future workforce of the allied health professions and as a result they provide a unique point of view on an area of practice that is a requirement of the workforce. The method used in this project enabled saturation to be reached, highlighting the quality of the research, furthermore the rigorous data analysis support the validity of the results of this study.

One limitation of this study is that the sample population are representative of only one year group of students in one university. Although this included a range of allied health professions, the timing and criteria for the focus groups excluded some AHP groups who were not in university at the time or had already graduated from the course; as a result not all AHP groups were represented in this study.

If this work is to be continued further focus groups with a wider sample must be conducted, such as all AHPs and across multiple universities. There was no attempt in this study to see if between student and graduation there was a change in perspective on this issue, which could be investigated. This study was conducted in 2016; by the time of publication the views and perceptions expressed here might well be outdated. Finally it must be asked how will a public health intervention agenda be integrated into what are already crowded curricula.

## Conclusion

This study has explored the perceptions of AHP students about their role in delivering BPHIA. The analysis revealed themes relating to 1) their understanding of public health, 2) the perceptions around their role and the role of others, 3) the barriers and challenges of integrating public health matters into their clinical role and 4) preparation for a public health role. In general AHP students had a positive view towards their role as public health messengers, but identified several barriers which impacted on their confidence and competence to deliver BPHIA effectively or optimally. Two key steps were identified around enhancing capacity to deliver public health messages. First, enhancing the integration of public health in the academic curriculum in order to optimise students understanding of how public health fits within patient-centred practice and to offer safe environment for students to engage with difficult conversations. Second, developing clinical placements which offer greater opportunity and support for developing skills in delivering BPHIA which are optimally tailored to patient need.

## Additional file

Additional file 1: Focus group topic guide. This file is the topic guide used to enable the focus group facilitator to explore relevant issues during the focus groups. (DOCX 16 kb)

## Abbreviations

AHP: Allied Health Professions
BPHIA: Brief public health interventions and advice
FG: Focus Group
HEI: Higher Education Institution
NHS: National Health Service
UK: United Kingdom
