# Supplementary material for: Healthcare students’ perceptions about their role, confidence and competence to deliver brief public health interventions and advice
Source: BMC Med Educ. 2018 May 24;18:114. doi: 10.1186/s12909-018-1224-0 (PMC5968571; doi:10.1186/s12909-018-1224-0)
Supplement: Supplementary file 1 — Focus group topic guide. This file is the topic guide used to enable the focus group facilitator to explore relevant issues during the focus groups. (DOCX 16 kb) [file 12909_2018_1224_MOESM1_ESM.docx]

**Additional file**

**Topic guide**

We might therefore begin with:

1) INTRODUCTION: Researcher introduces him/herself, reminds the group about the project, confidentiality (any issues that arise should not be discussed outside of the focus group) etc

2) Students identify themselves and their professions (so when we transcribe we know who is talking)

3) A general question about the interpretations of ‘public health’ shared by the students

1. What does public health mean to you
2. Can you give general examples of public health activities that you are aware of
3. What do you feel is the role of allied health professionals in delivering public health activities
4. What is the difference between activities such as public health campaigns and “brief public health interventions and advice”

4) What examples of “brief public health interventions and advice” have you witnessed?

1. Or if not witnessed, then perhaps are aware of?

5) How well do you that public health is supported by their professional groupings:

1. Are you awareness of any policy or practice guidelines from your professional body?

6) How well do you feel that public health has been covered by their education?

1. Can you give examples of modules which have covered public health issues?
2. Can you give examples of the types of information that has been covered during your courses
3. Can you give any examples where public health delivery might have been examined as part of your course work

7) How well do you feel that delivering public health interventions and advice was handled/encouraged by staff during your placement experience?

1. By you clinical mentor or other staff working on your placements
2. Did you yourself have the opportunity to deliver public health interventions or advice to patients
3. Were you encouraged to deliver public health interventions
4. Was there any particular factors that facilitated the process of delivering public health interventions or advice
5. Was there any particular factors that acted as a barrier

8) How empowered are they to talk about public health interventions and give advice.

1. What do you think is your role in delivering a variety of public health message? Why?
2. How knowledgeable do you feel around public health issues. In what ways do you feel knowledgeable? In what ways do you feel you lack knowledge?
3. How competent do you feel to deliver public health messages as part of your clinical practice? Why? What are your areas of competence, what areas would you feel less competent in.
4. How confident would you feel to approach a patient with a specific and obvious public health problem and discuss this with them;

- Physical activity, obesity, falls, smoking, alcohol, mental health
- If you do feel confident then what is it that helps you to feel confident in this situation?
- If you wouldn’t feel confident, then why do you feel that this is?

1. In a clinical setting where you are treating patients for one type of health condition do you think that there are any specific advantages? What might these be?
